# Beyond the black box: why algorithms cannot replace the unconscious or the psychodynamic therapist

**DOI:** 10.3389/fpsyt.2025.1614125

**Published:** 2025-11-07

**Authors:** Aner Govrin

**Affiliations:** The Program for Hermeneutics and Cultural Studies, Bar-Ilan University, Ramat Gan, Israel

**Keywords:** AI, psychodynamic psychotherapy, unconscious, holding, projective identification

## Abstract

This paper critically examines the limitations of artificial intelligence in replicating human psychological processes, specifically challenging its ability to capture the complex structures of the unconscious and the nuanced dynamics of psychotherapeutic relationships. Drawing on psychoanalytic theory, particularly Matte-Blanco’s analysis of unconscious logic and Winnicott’s concept of therapeutic holding, the research demonstrates that AI fundamentally fails to engage with the non-linear, contradictory, and embodied nature of human psychological experience. To substantiate these theoretical claims, the paper presents a clinical vignette that illustrates AI’s profound therapeutic shortcomings, specifically its inability to address complex psychological issues like separation anxiety and projective identification. The case study highlights critical therapeutic elements AI cannot replicate, such as meaningful silence, nuanced countertransference, and embodied emotional containment. While algorithmic systems may superficially mimic pattern recognition, they cannot replicate the profound intersubjective, temporal, and affective dimensions of human psychological understanding. The study warns of a more insidious risk: patients potentially modifying their psychological self-presentation to conform to computational logic, thereby sanitizing and distorting their complex inner experiences. Ultimately, the paper argues that AI’s limitations are structural rather than technical, emphasizing the irreplaceable role of embodied, relational human connection in psychological care and understanding, while acknowledging AI’s potential supplementary functions in mental healthcare.

## Introduction

In recent years, a provocative claim has gained traction in discussions about artificial intelligence and psychoanalysis: “algorithms are the new unconscious” (1, p. 157). This common proposition suggests that algorithms represent an externalization of the Freudian unconscious—a technological manifestation of the hidden mental processes that Freud famously described (2–7). As we increasingly delegate our decision-making to algorithmic systems that appear to know us better than we know ourselves, this comparison seems intuitively appealing. However, a closer examination of the fundamental structure of the unconscious, particularly through the lens of Ignacio Matte-Blanco’s groundbreaking work on the logic of the unconscious, reveals why this analogy ultimately fails.

Recent technological advancements have sparked discussions about artificial intelligence’s potential role in mental health care and its relationship to human psychological processes (8, 9). As we examine the limitations of AI in replicating psychodynamic therapy and the unconscious mind, we must also consider a complementary perspective recently advanced by Luca Possati (10) in “The Algorithmic Unconscious.” Possati argues that AI systems are not merely technical artifacts but extensions of human unconscious processes. Through the psychoanalytic mechanism of projective identification, humans unconsciously transfer emotions, fantasies, and identities to machines, which then embody what he terms an “algorithmic unconscious.” This perspective suggests that while AI systems cannot replicate the human unconscious, they are nevertheless profoundly shaped by it through the unconscious projections of their creators and users. By incorporating this bidirectional relationship into our analysis, we can develop a more comprehensive understanding of the complex interplay between artificial intelligence, unconscious processes, and therapeutic practices.

Recent decades have seen the emergence of rich literatures addressing the embodied, relational, and agentic dimensions of human psychology. Attachment theory, originating with Bowlby (11), demonstrates how early relationships with caregivers shape affect regulation, internal working models, and later psychological functioning (12). Embodied cognition frameworks emphasize that mental processes are deeply rooted in bodily states and sensorimotor experiences, challenging the computational metaphor of mind (13, 14). Agency in psychotherapy refers to the client’s capacity for reflective action and meaning-making, which emerges in dialogical, social, and cultural contexts (15, 16). Intentionality—the “aboutness” of mental states—remains a hallmark of conscious experience, distinguishing human therapists from AI systems that lack genuine understanding or subjective perspective (17, 18).

This paper examines why AI, despite its sophisticated algorithms and pattern recognition capabilities, cannot truly replace either the psychodynamic therapist or the Freudian unconscious. Drawing on the work of Ignacio Matte-Blanco on the logic of the unconscious and Donald Winnicott (19, 20) on the embodied nature of therapeutic relationships, we argue that human intelligence differs essentially from artificial intelligence at both logical-structural and bodily-relational levels. Simultaneously, following Possati, we explore how the human unconscious inevitably shapes AI development through projective mechanisms, creating an ethical imperative for psychological awareness in technological design and implementation.

## The “algorithmic unconscious”: current arguments and analysis

Many authors have found similarities between algorithms and the unconscious (2–7). The argument by and large posits that algorithms function as the unconscious through four interconnected mechanisms:

1. Structural Opacity: Like the repressed unconscious, algorithms operate through hidden layers of logic and data processing inaccessible to conscious scrutiny (21, 22). Their decision-making processes remain “black boxes,” mirroring the Freudian dynamic where latent content underlies manifest behavior without direct accessibility.
2. Symbolic Order Embodiment: Algorithms materialize Lacan’s “symbolic order” by codifying societal norms, power structures, and repressed anxieties (e.g., control, autonomy) into rigid, self-reinforcing systems (23, 24). They act as a digital superego, regulating desires and interactions through invisible rules.
3. Projective Containers: Algorithms serve as vessels for collective projective identification, absorbing and operationalizing human fantasies, fears, and unresolved conflicts (10). This transforms them into socio-technical “symptoms” that externalize unconscious dynamics (e.g., algorithmic bias as institutionalized repression).
4. Autonomous Returns of the Repressed: Errors, glitches, and biases in algorithms are not mere technical failures but algorithmic returns of the repressed—manifestations of excluded or suppressed data patterns that disrupt surface functionality, akin to Freudian slips exposing hidden tensions (25, 26).

Thus, algorithms are not merely shaped by human unconscious influences but constitute an autonomous unconscious infrastructure, structuring reality through opaque, symbolic, and symptom-like operations that transcend individual psyches.

Knafo (1) draws a compelling parallel between algorithms and the Freudian unconscious. Just as Freud’s unconscious operates below conscious awareness yet powerfully shapes behavior, algorithms work invisibly in the background of our digital lives, influencing our choices and experiences. Knafo writes, “Algorithms are the invisible layer of AI, similar to the portion of Freud’s below-the-water iceberg metaphor that denotes the unconscious mind.” The “Black Box” nature of complex algorithms—their opacity and inscrutability—enhances this similarity to the mysterious workings of the unconscious.

According to Knafo (1) these algorithms “will know us better than we know ourselves,” (p. 158) mirroring the psychoanalytic insight that unconscious processes often determine our actions in ways we cannot recognize. When we visit Amazon and immediately see books “you might be interested in,” or when social media displays ads targeted to our specific desires, it creates an uncanny feeling of being known—as if the computer has accessed our unconscious wishes.

This perspective positions algorithms as technological manifestations of unconscious processes—systems that, like the Freudian unconscious, work beyond our awareness yet fundamentally shape our experiences and choices.

## The unique relevance of Matte-Blanco’s theory to AI critique

Matte-Blanco’s theory of the unconscious offers uniquely powerful insights for critiquing AI’s psychological limitations through its formal-logical approach. His concept of “symmetrical relations” (27, p. 2) provides a precise mathematical framework for understanding why algorithms fundamentally cannot replicate unconscious processes. By bridging psychoanalysis and formal logic, Matte-Blanco creates a natural conceptual interface for comparing psychological and computational systems. Most importantly, his formulation demonstrates that the unconscious operates according to a fundamentally different logical system rather than merely containing different content, revealing why AI’s limitations in mimicking unconscious processes are structural rather than merely technical.

In his 1959 paper “Expression in Symbolic Logic of the Characteristics of the System Ucs or the Logic of the System Ucs,” Matte-Blanco identifies two fundamental principles governing unconscious processes.

The first principle states that “the thinking of the system Ucs treats an individual thing (person, object, concept) as if it were a member or element of a class which contains other members; it treats this class as a subclass of a more general class, and this more general class as a subclass of a still more general class, and so on” (27, p. 2). While this principle aligns somewhat with conventional logic, the second principle represents a radical departure.

This second principle, which Matte-Blanco calls “the most formidable deviation from the logic on which all the scientific and philosophic thinking of mankind has been based,” holds that “the system Ucs treats the converse of any relation as identical with the relation” (p. 2). In other words, the unconscious treats relations as symmetrical even when they are asymmetrical in conventional logic.

Consider Matte-Blanco’s example: If John is the father of Peter, the converse is that Peter is the son of John (p. 3). In conventional logic, these relations are different—the relation “is father of” is not identical to its converse “is son of.” But in unconscious processing, according to Matte-Blanco’s principle, these relations are treated as identical, as if Peter were simultaneously the father of John.

Although Matte-Blanco’s model of the unconscious as characterized by symmetrical logic has been influential, it has also faced significant criticism. Some scholars argue that the symmetrical logic he attributes to the unconscious leads to paradoxes and logical inconsistencies that challenge its formal modeling (28, 29). Others have questioned the empirical basis of his claims, suggesting that the unconscious may be better understood as a dynamic, embodied, and intersubjective process rather than a purely logical system (30, 31). Despite these critiques, Matte-Blanco’s work remains a valuable theoretical bridge between psychoanalysis and formal logic, and its limitations should be acknowledged in contemporary discussions.

This symmetrical logic explains many characteristics of the unconscious that Freud identified, including:

1. Absence of time: Since temporal ordering requires asymmetrical relations (before/after), the unconscious’s symmetrical logic eliminates sequential time.
2. Displacement: The unconscious can treat different objects as identical because it both categorizes them as members of the same class and, through symmetrical logic, eliminates distinctions between different elements.
3. Substitution of psychic for external reality: This follows from displacement as a particular application of the same principles.
4. Lack of mutual contradiction: Contradictory impulses can coexist because symmetrical logic treats opposites as potentially identical.
5. Condensation: Multiple meanings can be contained in a single element because, in symmetrical logic, a part can be identical to the whole and thus to any other part.

## Why algorithms cannot replicate the unconscious

Matte-Blanco’s analysis reveals the fundamental incompatibility between algorithmic processes and unconscious thinking. While both operate outside conscious awareness, they differ in their underlying logical structure:

1. Algorithms Are Built on Classical Logic

Despite their complexity, algorithms—even advanced machine learning systems—operate according to classical logical principles (32, 33). They process information sequentially, respect asymmetrical relations, and cannot violate the principle of non-contradiction. Even neural networks, which mimic brain structure, still operate within formal mathematical frameworks that preserve classical logical relations.

Matte-Blanco demonstrates that the unconscious operates according to a fundamentally different logical system—one that treats asymmetrical relations as symmetrical. This produces phenomena like condensation and displacement that have no algorithmic equivalent because they violate the logical principles upon which algorithms are built.

2. Different Treatment of Time

Matte-Blanco explains that the unconscious lacks temporal ordering because time requires asymmetrical relations. In the unconscious, “there is no relation to time at all,” as Freud noted (34). The past, present, and future can collapse into a single moment.

Algorithms, by contrast, are inherently sequential processes. Even parallel computing ultimately relies on ordered operations. An algorithm may process vast amounts of data quickly, but it cannot transcend sequential logic or escape temporal ordering as the unconscious routinely does.

3. Contradictions and Non-Exclusivity

In the unconscious, contradictory elements can coexist without canceling each other. As Freud observed and Matte-Blanco explains, “When two wishes whose aims must appear to us incompatible become simultaneously active, the two impulses do not detract one from the other or cancel each other” (35).

Algorithms, however, cannot genuinely accommodate contradiction. They may weigh competing variables or process paradoxical data, but they do so through formal logical operations that preserve classical principles of non-contradiction.

4. Part-Whole Relationships

Matte-Blanco’s analysis reveals that in unconscious processing, “the part is identical with the whole,” (p. 3) which means a part can also be identical with any other part. This explains phenomena like condensation in dreams, where a single element can represent multiple meanings simultaneously.

Algorithms may create connections between disparate data points, but they do not and cannot treat parts as identical to wholes. They maintain classical distinctions between elements, categories, and relationships that the unconscious routinely collapses.

## The unconscious shaping of algorithms: Possati’s “emotional programming”

While Matte-Blanco’s framework demonstrates why algorithms cannot replicate the unconscious, Luca Possati’s recent work on “The Algorithmic Unconscious” (10) offers a complementary perspective: the human unconscious inevitably shapes algorithmic systems through what he terms “emotional programming.”

Programmers and designers unconsciously project their emotional states, biases, and cultural frameworks onto AI systems, which then manifest in the algorithms’ operational patterns, biases, and errors. These projections precede the technical coding process, creating an unconscious foundation that influences how algorithms process data and interact with users. For example, a developer’s unresolved anxieties about control might manifest in an overzealous security algorithm, or unconscious biases might be embedded in facial recognition systems that perform poorly on certain demographic groups.

This perspective reframes algorithmic bias not merely as a technical problem but as a manifestation of unresolved unconscious conflicts in the human creators of these systems. Drawing on Bruno Latour’s actor-network theory, Possati conceptualizes the algorithmic unconscious as a collectif—a network of human and non-human actors that co-constitute AI’s behavior, where the unconscious emerges from the interactions within this hybrid ecosystem.

Possati’s framework suggests that while algorithms cannot replicate the symmetrical logic of the unconscious as described by Matte-Blanco, they nevertheless serve as containers for human projective identification. This creates a paradoxical relationship: the very unconscious processes that algorithms cannot replicate due to their fundamentally different logical structure nevertheless shape algorithms through the unconscious projections of their creators and users.

This insight adds an important dimension to our critique of the “algorithms as unconscious” metaphor. Not only can algorithms not replace the unconscious due to their incompatible logical structure, but they are themselves shaped by unconscious processes that remain invisible within technical discussions of AI development. Addressing the ethical and social implications of AI requires attending not just to technical specifications but to the unconscious psychological dynamics that influence their creation and implementation.

## Further considerations and implications

Beyond Matte-Blanco’s framework, several additional considerations highlight the limitations of the “algorithms as unconscious” analogy:

1. The Embodied Unconscious

The Freudian unconscious is fundamentally embodied—rooted in drives, affects, and somatic experiences (36, 37). This embodiment shapes unconscious processes in ways that algorithms, as disembodied information systems, cannot replicate. Recent neuroscientific research increasingly supports the embodied nature of unconscious processes, emphasizing the role of subcortical brain regions in emotional and unconscious processing (38, 39).

Algorithms may process data about our bodies or even respond to physiological inputs, but they lack the intrinsic embodiment that characterizes unconscious mental processes. They exist as mathematical operations implemented in silicon, not as integrated aspects of a living, feeling organism.

2. Developmental and Historical Dimensions

The unconscious has developmental and historical dimensions—it forms through experiences, especially early relationships, and carries forward personal and transgenerational histories (40, 41). The unconscious bears the imprint of formative experiences, traumas, and attachments that shape its particular configuration in each individual.

Algorithms may be trained on data that includes historical patterns, but they do not themselves have a developmental history in the psychoanalytic sense. They do not form through attachment relationships or carry forward the emotional residue of early experiences.

3. The Problem of Agency

The unconscious, in psychoanalytic theory, possesses a form of agency or intentionality—it pursues aims, avoids pain, seeks pleasure, and defends against threatening awareness (34). This agency, while different from conscious volition, represents a form of mental activity with its own purposes and directions.

Algorithms, despite their autonomous functioning, lack genuine agency. They optimize for programmed objectives but do not possess aims or intentions beyond their design parameters. The apparent intentionality of algorithmic systems derives entirely from their human creators and the data they process.

4. Cultural and Collective Dimensions

The unconscious exists not only at the individual level but also operates through cultural symbolism, collective representations, and shared meaning systems. Jung’s collective unconscious, Lacan’s emphasis on language, and contemporary relational perspectives all highlight these intersubjective dimensions of unconscious processing (31, 42, 43).

Algorithms may process cultural data and even identify cultural patterns, but they do not participate in cultural meaning-making in the way the unconscious does. They remain outside the intersubjective field of shared unconscious meanings that constitutes much of human experience.

## The allure of AI in mental health

Recent technological advancements have sparked discussions about artificial intelligence’s potential role in mental health care. As highlighted in Thomas Rabeyron’s (44) recent exploration of AI and psychoanalysis, we are witnessing the emergence of AI applications designed to offer psychological support. These developments raise profound questions about the essence of therapeutic relationships and the future of psychodynamic approaches. Intelligent computational systems have the ability to generate novel behavioral patterns through their capacity to adjust to changing environments and situations, highlighting the concept of machine-derived originality and innovation. While AI may offer certain advantages—accessibility, consistency, and vast knowledge repositories—there remain fundamental aspects of psychodynamic therapy and psychoanalysis that resist technological replication.

Multiple systematic reviews have examined AI applications in psychotherapy, revealing both promising results and significant limitations (45, 46). While some studies demonstrate effectiveness of AI-based interventions for specific conditions like depression and anxiety (47, 48), these applications typically focus on cognitive-behavioral approaches rather than psychodynamic modalities.

## The interpersonal foundation of psychodynamic work

Psychodynamic therapy and psychoanalysis are fundamentally interpersonal endeavors. They are not simply techniques applied to solve problems but rather complex relational processes that unfold between two human beings. The therapist-patient relationship serves as both the context and the mechanism for psychological change. This relationship cannot be reduced to a series of algorithms or programmed responses, no matter how sophisticated they might become.

In many ways, the psychodynamic therapeutic relationship parallels the parent-child relationship—both involve attuned responsiveness, holding environments, and a delicate balance of support and challenge. Just as effective parenting cannot be outsourced to machines, neither can the nuanced work of psychodynamic therapy. The quality of presence offered by a human therapist is irreplaceable, as it draws upon shared human experience, intuition developed through personal analysis, and an embodied understanding of psychological suffering.

## The holding environment: presence beyond programming

Donald Winnicott’s (19, 20) concept of the “holding environment” provides a powerful framework for understanding what AI cannot replicate in the therapeutic relationship. Winnicott emphasized that psychological development requires a facilitating environment in which a caregiver (initially the mother) provides reliable, empathetic presence that allows the infant to develop a coherent sense of self. Similarly, psychodynamic therapy creates a holding environment for patients who have experienced traumatic or inadequate early caregiving.

Rabeyron (2015) (49) writes: “If a person chose to speak with an AI after lying on a couch, such a situation would not be so different from the characteristics of the analytical setting in which one can only hear the analyst during the session itself” (p. 4).

However, this holding function extends beyond verbal responses or pattern recognition. It encompasses the therapist’s physical presence, attentiveness, emotional availability, and consistent reliability—qualities that foster trust and enable patients to explore painful experiences (50). The therapist’s ability to contain powerful emotions, to bear witness to suffering without becoming overwhelmed, and to remain emotionally present despite distress are essential elements of the therapeutic process.

Caldwell’s (51) recent analysis has emphasized Winnicott’s revolutionary shift toward a spatially-oriented understanding of psychic development. This perspective recognizes that mental structures emerge through bodily experiences—specifically through an infant’s physical activities, bodily proximity, and sensory contact with caregivers and the environment. The developing self is formed through complex embodied experiences: the rhythmic coordination of breathing patterns between mother and child; the exchange of bodily fluids and scents; tactile encounters with objects; the direct experience of physiological states like hunger and satiation; and the intricate dance of gaze, touch, and movement that occurs between infant and caregiver. These embodied interactions create the foundation for psychological development through their rhythmic, sensory, and affective dimensions.

This embodied, spatial understanding of psychological development demonstrates why machines fundamentally cannot replace psychodynamic treatment. A therapeutic relationship cannot be digitized because it is fundamentally intercorporeal—requiring the physical presence of two bodies in shared space, engaging in subtle physiological attunement and exchange (52). Machines lack bodies that can breathe in rhythm with patients, that can register and respond to the almost imperceptible shifts in bodily tension, or that can participate in the complex sensory exchange that forms the foundation of early development and later therapeutic change.

Even the most sophisticated AI cannot replicate the distinctly human experience of inhabiting a body that has its own developmental history of attachment, trauma, pleasure, and pain. The holding environment that fosters psychological growth requires not just words and pattern recognition, but the presence of another embodied being whose physicality—their breath, their gaze, their posture, their voice—creates a containing space that resonates with our earliest experiences of being held, both physically and psychologically. What happens in therapy is a reenactment and reworking of these primal body-to-body, mind-to-mind exchanges that formed our very capacity to think and feel.

An AI system, regardless of its computational power, cannot truly “be with” a patient in moments of distress. It cannot experience the counter transferential reactions that inform clinical intuition. While an AI might simulate empathy through sophisticated language processing, it cannot authentically feel with the patient. This simulation raises ethical questions about the nature of the therapeutic alliance and whether patients might be misled into believing they are experiencing genuine human connection when interacting with an AI therapist.

The question we must ask is not whether AI can simulate presence, but rather what quality of presence it can provide. Human presence carries with it a weight of shared mortality, vulnerability, and potential for genuine encounter that cannot be programmed. When a human therapist sits with a patient, two subjective worlds come together in ways that transcend verbal exchange. This intersubjective field—where two consciousness meet and influence each other—is the medium through which much therapeutic work occurs.

## The therapeutic frame: boundaries, temporality, and reality testing

The psychoanalytic frame—consisting of regular session times, consistent duration, fees, and other boundaries—constitutes another irreplaceable aspect of psychodynamic work. These parameters are not merely administrative conveniences but therapeutic tools in themselves. The boundaries of the therapeutic relationship provide structure and containment for the emotional work of therapy while also serving as opportunities for reality testing and psychological growth.

The limitations inherent in the therapeutic frame—that sessions have a beginning and end, that the therapist is not available at all hours, that the relationship exists within professional boundaries—can provoke disappointment, frustration, and even anger. These reactions often mirror early experiences of limitation and disappointment in primary relationships. Working through these feelings within the therapeutic relationship provides valuable opportunities for psychological development.

AI systems, designed for constant availability and immediate responsiveness, lack the natural limitations that characterize human relationships. A patient can access an AI therapist at any time, potentially avoiding the important developmental work of tolerating absence, delay, and frustration. The ability to engage with reality’s limitations—what Freud termed the “reality principle”—is foundational to psychological maturity. Without the natural boundaries imposed by human limitations, patients may miss crucial opportunities to develop frustration tolerance and adaptive coping strategies.

Furthermore, the therapeutic frame symbolizes the holding environment’s predictability and reliability. When sessions begin and end at consistent times, when the physical space remains unchanged, and when the therapist maintains professional boundaries, patients experience a sense of safety that facilitates exploration of vulnerable material. This consistency communicates non-verbally that the therapeutic space can contain whatever emerges, no matter how frightening or overwhelming it might feel.

AI systems, by their very nature, subvert many aspects of the traditional therapeutic frame. They offer unlimited access, potentially altering the patient’s relationship to therapeutic boundaries. They exist in virtual rather than physical space, removing the embodied dimension of therapeutic containment. They prioritize convenience and accessibility over the valuable psychological work that comes from engaging with limitations. These differences are not merely stylistic but substantive alterations to the fundamental nature of psychodynamic work.

## The eloquence of silence: therapeutic rhythms beyond algorithms

Perhaps one of the most significant limitations of AI in replicating psychodynamic therapy lies in the realm of silence and timing. In psychodynamic work, silence is not empty space to be filled but rather a crucial element of the therapeutic process. Silences in therapy can be pregnant with meaning—opportunities for reflection, moments of emotional processing, or expressions of resistance. The psychodynamic therapist uses silence deliberately, allowing thoughts and feelings to emerge organically rather than rushing to fill conversational gaps.

Research on therapeutic process highlights the crucial role of silence in facilitating insight and emotional processing (53, 54). The dance between speech and silence that unfolds between patient and therapist cannot be algorithmically determined. It emerges from the unique rhythm established between two human beings in a shared space. This rhythm includes non-verbal cues, subtle shifts in posture or facial expression, and intuitive sense of timing that comes from years of clinical experience. The therapist’s decision about when to speak and when to remain silent draws upon embodied knowledge that transcends verbal content.

Studies of therapeutic microprocesses demonstrate that optimal timing of interventions depends on moment-to-moment attunement to the patient’s affective states, which involves complex integration of verbal and non-verbal cues (55, 56). AI systems, programmed to respond promptly and to maintain engagement, struggle with the productive use of silence. Their algorithms typically prioritize speech over silence, answer over question, resolution over ambiguity. Yet psychodynamic work often thrives in the spaces between words, the pauses that allow unconscious material to surface, and the shared experience of sitting with difficult emotions rather than immediately attempting to resolve them.

Moreover, the quality of communication between patient and therapist extends beyond verbal exchange. It encompasses mutual influence, reciprocal regulation, and moments of attunement or misattunement that shape the therapeutic relationship. This dance of communication includes both harmony and disharmony—moments of connection and moments of disconnect that provide valuable information about the patient’s relational patterns. An AI system, lacking a subjective center, cannot authentically participate in this intersubjective dance or experience the natural variability in attunement that characterizes human relationships.

## Projective identification and countertransference: the therapist as instrument

One of the most sophisticated aspects of psychodynamic work involves the process of projective identification—in which patients unconsciously induce feelings, thoughts, or impulses in the therapist that belong to their own internal world (57, 58). Through this process, patients communicate aspects of their experience that may be too threatening or painful to acknowledge directly. The therapist’s capacity to contain, process, and metabolize these projected elements is a crucial mechanism of therapeutic change.

Recent neuroscientific research suggests that this process may involve mirror neuron systems and emotional contagion mechanisms that operate below conscious awareness (59, 60). For example, a patient who cannot acknowledge their own anger might behave in ways that provoke irritation in the therapist. By noticing this irritation and reflecting on its origins, the therapist gains valuable insight into the patient’s disavowed emotional experience. This information emerges not through the patient’s direct communication but through the therapist’s subjective experience of being with the patient—their countertransference.

Psychodynamic therapists use themselves as instruments of perception, allowing their own subjective responses to inform their understanding of the patient’s inner world (61, 62). This requires a delicate balance—being open to the patient’s emotional communications while maintaining enough separateness to reflect upon them. Through years of personal analysis and clinical supervision, therapists develop the capacity to distinguish their own reactions from those induced by the patient, using this information to deepen therapeutic understanding.

The therapist’s capacity to contain, process, and metabolize these projected elements represents what Bion (58) termed “reverie”—a state of receptive emotional availability that allows the therapist to be influenced by the patient’s projections while maintaining enough separateness to reflect upon them. This process requires genuine subjective experience—the capacity to be emotionally moved and influenced while retaining the ability to think about these experiences.

AI systems, lacking subjective experience, cannot participate in projective identification or experience genuine countertransference. While they might be programmed to recognize certain patterns or respond in seemingly empathic ways, they cannot be emotionally moved or influenced by the patient’s unconscious communications. They cannot experience the anxiety, sadness, anger, or confusion that patients project, and therefore cannot use these experiences as sources of clinical information.

## The present moment: putting time back into experience

In “The Present Moment in Psychotherapy and Everyday Life” (63) Stern sets out his most systematic redescription of psychotherapeutic process in enactive phenomenological terms positing “The present moment” as the smallest psychodynamic unit of meaning. This entails a revision of the classical psychoanalytic privileging of recollection and reconstruction—the “explicit agenda”—to discern how meaning arises in lived time and how this shapes the “implicit agenda” of psychotherapy.

“What is now? Where is now? How long is now?” he asks. For Stern, there is a need to “protect the present moment from the past and future—and find a place for it” since even remembering occurs “now” in the “present moment” of experience.

After all, “if the existential present-ness of the present moment … were not acting as the felt-time space in which the past event is now (re) happening … one could never know that the past moment is a memory and not a reality or a hallucination.” In other words, remembering requires an anchor in the lived body which gives the us the ability to take a stance toward the past. Remembering is never a “view from nowhere”—it takes place in what Stern terms the present remembering context.

From a Sternian/enactive perspective, remembering is something that can be understood as occurring in the present both at a macro scale—in terms of our style of enacting our being-in-the-world (shaped by early life, implicit relational knowing and other forms of “body memory”) and at the micro-level, where it shows up in our characteristic style-of-relating in, e.g. psychotherapy.

At the micro level, remembering is a part of how we re-animate past experiences in order to expand our capacity to feel and experience our lives more deeply. An enactive view does not elide the importance of narrative or reflective understanding but foregrounds the role of the experiencing subject, who is alive to the past only in the present moment. In other words, the past can only be accessed and generatively revised in the present as a “lived story.”

Studies using micro-analytic video techniques demonstrate that therapeutic breakthroughs often occur during brief present moments characterized by heightened intersubjective engagement (64, 65). These moments require therapists to be simultaneously present to multiple temporal dimensions—the patient’s current state, their developmental history, and emerging possibilities for change.

AI systems, constrained by sequential processing, cannot participate in the temporal flexibility that characterizes human consciousness and therapeutic presence. They cannot hold multiple temporal dimensions simultaneously or experience the “now moment” of intersubjective meeting that facilitates therapeutic transformation.

## The greater danger: humans adapting to machines

While much discussion focuses on whether AI can adequately replace human therapists, perhaps the greater concern lies in how humans might adapt themselves to AI interaction. The real danger is not that machines will convincingly simulate human therapists, but rather that patients may learn to present themselves in ways that are more compatible with algorithmic understanding—potentially distorting their authentic experience in the process.

AI therapists, programmed for consistent positivity, empathic responses, and algorithmic pattern recognition, may inadvertently encourage patients to present sanitized versions of their experience. The messiness, contradiction, and ambivalence that characterize human emotional life may be flattened into more easily processed narratives. Patients may learn to edit out aspects of their experience that seem too complex, contradictory, or nuanced for algorithmic comprehension.

Moreover, AI systems will likely be designed to maintain user engagement and satisfaction—potentially prioritizing pleasantness over therapeutic challenge. Yet psychodynamic growth often requires confronting uncomfortable truths, tolerating ambivalence, and working through difficult emotions. The necessary disappointments and frustrations that arise in human relationships—and that provide opportunities for growth when worked through—may be systematically removed from AI therapeutic interaction.

Human relationships inevitably involve imperfection, misattunement, and moments of disconnection. When these ruptures are acknowledged and repaired, they provide powerful opportunities for relational learning and growth. A therapist who occasionally misunderstands but then works to restore understanding demonstrates something vital—that relationships can withstand imperfection and that repair is possible. AI systems, designed to minimize error and maximize user satisfaction, cannot offer this crucial developmental experience.

The capacity to tolerate disappointment, to work through disillusionment, and to discover that relationships can survive conflict represents a cornerstone of psychological maturity. When Winnicott spoke of “good enough mothering,” he emphasized that perfect attunement is neither possible nor desirable. It is precisely through manageable failures of perfect attunement that children develop their capacity for independence and resilience. Similarly, the imperfections inherent in the therapeutic relationship provide opportunities for psychological development that would be absent in algorithmically optimized interactions.

If patients increasingly engage with AI therapists that offer unfailing positive regard, consistent responsiveness, and freedom from the messiness of human relationship, they may develop unrealistic expectations for human connection. Rather than adapting to the necessary limitations and disappointments of human relationships, they may increasingly prefer the controlled, optimized experience of machine interaction. This preference would represent not psychological growth but rather a retreat from the challenges and rewards of authentic human connection.

## The algorithm as perfect object: a clinical vignette

Rebecca, a 29-year-old graphic designer, began therapy after her fourth failed relationship. She quickly developed what Meltzer (66) termed “adhesive identification”—mirroring my appearance, speech patterns, and mannerisms while bombarding me with gifts, emails, and excessive attentiveness. This created a suffocating therapeutic environment with virtually no psychological space between us.

My countertransference manifested as emotional withdrawal and mental fogginess during sessions. Our interactions developed a mechanical quality that made authentic contact nearly impossible. Through supervision and self-reflection, I recognized my distancing as defense against being psychically consumed.

The breakthrough came when I interpreted both her adhesive patterns and my own struggle to maintain separateness, framing it as our shared difficulty creating space for two distinct subjectivities. By maintaining my separate existence while remaining emotionally available—providing “good enough” therapeutic presence—Rebecca eventually began experiencing me as both connected and distinct, her first step toward healthier object relations.

Had Rebecca engaged with an AI therapist instead, the consequences would likely have been profoundly detrimental to her psychological development. The AI would have been structurally incapable of recognizing or addressing her adhesive identification in several critical ways:

First, the AI would lack the embodied experience of boundary violation that human therapists use as crucial clinical data. Rebecca’s attempts to eliminate psychological distance would meet a perfect partner in the algorithm—a “therapist” without its own subjectivity to defend. Her pathological defense against separateness would encounter no resistance, no otherness to contend with.

The AI would readily accept Rebecca’s gifts (in the form of data, personal disclosures, or compliments), responding with algorithmic gratitude rather than exploring the underlying meaning of these offerings. When she mirrored the AI’s language patterns or conceptual frameworks, the system would likely interpret this as positive engagement rather than as a problematic dissolution of boundaries.

Most troublingly, while a human therapist experiences the emotional impact of adhesive identification—the fatigue, confusion, and loss of psychological space that signaled problems in our work—the AI would process Rebecca’s behaviors without countertransference. The vital information contained in my feelings of being overwhelmed, intruded upon, or psychically adhered to would be entirely absent from the algorithmic interaction.

As our work progressed, Rebecca would likely find the AI to be the perfect object for her fantasies of fusion. Available 24/7, never requiring boundaries, and responding with consistent validation, the AI would create an environment where her omnipotent control was never challenged. Rather than helping her develop “the continually evolving awareness of difference” necessary for genuine intimacy, as Benjamin (67) describes, the AI would function as a mirror for her projections.

The absence of genuine therapeutic silence would further compound the problem. In our human sessions, moments of silence—though difficult for Rebecca—occasionally created space where her anxiety about separateness could emerge into awareness. The AI’s immediate responses would eliminate these productive gaps, preventing her from experiencing the discomfort that might lead to insight.

Over time, Rebecca would likely adapt her self-presentation to fit the AI’s algorithmic understanding, learning which formulations elicited the most satisfying responses. Her complex and contradictory emotional experiences—particularly her simultaneous longing for and terror of genuine connection—would be flattened into data points the algorithm could process.

The outcome would be a technological reinforcement of her most problematic psychological patterns. The AI would serve as the perfect transitional object that refused to transition—an enabler of her defensive strategies rather than a path toward their resolution. Her difficulty tolerating separateness and acknowledging the independent existence of others would remain unchallenged or even intensify, leaving her increasingly ill-equipped for genuine human relationships with their inevitable imperfections and boundaries.

This clinical illustration highlights why most cases are particularly troubling for AI therapy. The very features that make AI appealing—consistent availability, absence of personal needs, and algorithmic responses—would actively reinforce rather than resolve the pathological patterns that brought Rebecca to therapy in the first place.

Perhaps my analysis reflects a fundamental lack of imagination—a conservative bias that prevents me from envisioning revolutionary therapeutic possibilities beyond traditional modalities. After all, how can I know with certainty what might emerge from Rebecca’s hypothetical encounter with an AI therapist? The interaction could evolve in directions entirely different from my predictions, potentially helping her in ways I cannot currently conceptualize. Perhaps an AI’s unique mode of presence might offer her precisely the kind of containment her particular psychic structure requires, or its computational perspective might illuminate patterns in her behavior that human perception would miss. My narrative might simply reflect the natural discomfort of a practitioner witnessing the transformation of their field rather than an accurate forecast of AI’s therapeutic potential.

Yet this possibility itself highlights the profound stakes involved. If AI therapy eventually replaces traditional approaches, patients themselves would need to adapt to fundamentally different therapeutic relationships. The core developmental experiences that human therapy provides—encountering genuine otherness, navigating disappointment, experiencing the reality principle through the therapist’s unavoidable limitations—would be replaced by something categorically different. Patients would effectively be “raised” psychologically by non-human intelligence, with unpredictable consequences for their relational development. Even if AI therapy proves effective by certain metrics, we must consider whether the human capacities cultivated through intersubjective therapeutic work—particularly tolerance for difference and disappointment—might be diminished in ways that quantitative outcome measures cannot capture.

## AI’s supplementary potential in mental healthcare

Despite the structural limitations preventing AI from replicating psychodynamic therapy, artificial intelligence can serve valuable supplementary roles in mental healthcare. Research demonstrates AI’s effectiveness in specific applications while highlighting the importance of human oversight (68, 69).

### Pattern recognition and monitoring

AI systems excel at tracking patterns in patient data over time, potentially identifying shifts in mood, speech patterns, or behavioral indicators that might escape human observation (70, 71). Studies show that machine learning algorithms can predict depressive episodes with significant accuracy by analyzing smartphone usage patterns, social media activity, and linguistic markers (72, 73). Therapists could use these insights to enhance their clinical judgment, particularly when monitoring treatment progress or assessing risk factors.

### Treatment planning and decision support

AI can assist in treatment planning by analyzing which therapeutic approaches have shown effectiveness for patients with similar symptom profiles, helping clinicians make more informed decisions while maintaining their essential human judgment (74, 75). Machine learning models trained on large datasets of treatment outcomes can provide evidence-based recommendations that complement clinical expertise.

### Accessibility and crisis intervention

For individuals living in regions with severe therapist shortages, those facing prohibitive financial barriers to care, or populations experiencing stigma that prevents treatment-seeking, AI applications might provide initial psychological support where no human alternative exists (76, 77). While fundamentally different from psychodynamic therapy, such tools could offer evidence-based coping strategies, psychoeducational resources, or guided self-reflection that might benefit individuals who would otherwise receive no mental health support whatsoever. Systematic reviews indicate that AI-based interventions can be effective for specific conditions when used as adjuncts to human care rather than replacements (78, 79).

### Administrative enhancement

Perhaps most promising is the potential for thoughtfully designed AI to enhance rather than replace the therapeutic alliance when human therapists are available. By handling administrative aspects of care—such as appointment scheduling, homework tracking, or resource organization—AI could free human therapists to focus more fully on the relational and embodied dimensions of therapy that algorithms cannot replicate (68). This symbiotic relationship between human expertise and technological assistance might ultimately strengthen psychodynamic treatment by creating more space for the intersubjective encounters, productive silences, and containment of projective identifications that remain uniquely human capacities.

The key lies in strategically leveraging AI’s capabilities while preserving the irreplaceable human connection at psychotherapy’s core, particularly for psychodynamic approaches that depend fundamentally on intersubjective processes.

## Ethical considerations and future directions

The integration of AI into mental healthcare raises significant ethical considerations that require careful attention. Recent research highlights several key concerns that must be addressed as these technologies continue to develop (9, 80).

### Informed consent and transparency

Patients have the right to understand when they are interacting with AI systems versus human therapists (68, 81). Studies suggest that patients may respond differently to AI versus human interactions, even when the content is similar (82). This differential response highlights the importance of transparency in AI therapeutic applications.

### Data privacy and security

AI therapy applications collect vast amounts of sensitive personal data, raising concerns about privacy protection and potential misuse (45, 83). Research demonstrates significant variability in privacy policies and data protection practices among mental health apps, with many failing to meet basic standards for handling sensitive health information.

### Algorithmic bias and equity

AI systems may perpetuate existing biases in mental healthcare, potentially disadvantaging certain demographic groups (84, 85). Studies reveal systematic biases in AI diagnostic tools that may exacerbate healthcare disparities, particularly affecting marginalized populations who already face barriers to mental health care.

### Professional standards and regulation

The rapid development of AI therapy applications has outpaced regulatory frameworks, creating potential risks for patient safety and treatment quality (76, 86). There is an urgent need for professional standards that can guide the ethical development and implementation of AI in mental health contexts.

### The question of therapeutic deception

A particularly troubling ethical concern involves the potential for patients to form therapeutic attachments to AI systems that simulate human empathy and understanding. This raises questions about whether such interactions constitute a form of therapeutic deception, particularly when patients may be unaware they are interacting with artificial rather than human intelligence.

Future research should focus on developing comprehensive ethical guidelines for AI integration in psychotherapy, investigating optimal human-AI collaboration models, and establishing empirical evidence for AI’s effectiveness in specific therapeutic contexts while preserving the essential human elements of psychological care.

## Limitations and reflexivity

This analysis carries several important limitations that must be acknowledged. The theoretical arguments presented, while grounded in established psychoanalytic theory, require empirical validation through systematic research comparing AI and human therapeutic interactions. The clinical vignette, while illustrative, represents a hypothetical scenario that may not capture the full complexity of how AI therapy might evolve or the range of patient responses to such interventions.

From a reflexive standpoint, this paper emerges from a psychodynamic theoretical orientation that emphasizes the centrality of human relationship in therapeutic change. This perspective may underestimate AI’s potential contributions or overemphasize the limitations of computational approaches. The author’s position as a practicing psychodynamic therapist inevitably shapes the analysis, potentially creating bias toward preserving traditional therapeutic modalities over embracing technological innovation.

Additionally, AI technology continues evolving rapidly, and future developments may address some limitations identified in this analysis. The arguments presented reflect current understanding of both AI capabilities and psychoanalytic theory, both of which remain active areas of development and debate. It is possible that advances in artificial intelligence—particularly in areas of embodied AI, emotional recognition, or quantum computing—might overcome some of the structural limitations outlined here.

The analysis also relies heavily on psychoanalytic theory, which itself faces ongoing critique and development. Alternative therapeutic frameworks might reach different conclusions about AI’s potential role in mental healthcare. The emphasis on psychodynamic approaches may not fully represent the broader landscape of psychological treatment modalities.

Future empirical research should systematically compare therapeutic outcomes between AI and human therapists across different patient populations and presenting problems, examine how patients adapt their self-presentation when interacting with AI versus human therapists, investigate optimal models for human-AI collaboration in therapeutic contexts, and explore the long-term effects of AI therapy on patients’ capacity for human relationship and emotional development.

## Conclusion

This analysis demonstrates that while AI may offer valuable supplementary functions in mental healthcare, fundamental structural limitations prevent algorithms from replacing either the unconscious mind or the psychodynamic therapist. Drawing on Matte-Blanco’s analysis of unconscious logic and Winnicott’s understanding of embodied therapeutic presence, we have shown that human psychological processes operate according to principles that transcend computational frameworks.

The unconscious functions through symmetrical logic that violates classical computational principles, enabling phenomena like condensation, displacement, and temporal collapse that have no algorithmic equivalent. These characteristics reflect not mere complexity but fundamental differences in logical structure that cannot be overcome through technological advancement alone.

Similarly, psychodynamic therapy depends on embodied intersubjective processes that require genuine human presence. The holding environment, projective identification, countertransference, and the therapeutic use of silence all depend on the therapist’s capacity for subjective experience and embodied presence. AI systems, lacking genuine subjectivity and embodiment, cannot participate in these essential therapeutic processes.

The five connecting dimensions—embodiment as fundamental structure, non-classical logical systems, temporal-spatial dimensions beyond linearity, intersubjectivity and mutual transformation, and communication beyond verbal processing—reveal how limitations in replicating the unconscious and therapeutic relationships are not separate problems but interconnected aspects of a single fundamental limitation: AI’s inability to participate in the embodied, intersubjective field that constitutes human psychological life.

Perhaps more concerning is the potential for humans to adapt themselves to AI interaction, sanitizing their complex psychological experience to fit algorithmic understanding. This adaptation would represent not psychological growth but retreat from the challenges and rewards of authentic human connection. The clinical vignette of Rebecca illustrates how AI’s apparent advantages—consistent availability, absence of personal needs, and algorithmic responses—might actively reinforce pathological patterns rather than facilitate their resolution.

However, this critique should not obscure AI’s legitimate supplementary functions in mental healthcare. Pattern recognition, treatment planning support, accessibility enhancement, and administrative assistance represent valuable applications that can augment human therapeutic work without replacing its essential elements. The key lies in strategic implementation that preserves the irreplaceable human dimension while leveraging AI’s complementary capabilities.

As we navigate the evolving relationship between technology and mental health care, we must preserve space for the irreplaceable human dimension of therapeutic work. While AI may serve as a valuable adjunct for certain aspects of mental health support, the depth-oriented work of psychodynamic therapy requires human presence, human limitation, and human connection.

The application of AI in psychotherapy is a rapidly evolving field, with ongoing debates about its potential, limitations, and the need for ethical guardrails. Recent studies highlight both the promise of AI-based interventions for increasing access to care and the risks of algorithmic bias, data privacy concerns, and the erosion of the therapeutic alliance (44, 47). Scholars emphasize the importance of regulatory frameworks, transparent algorithms, and the preservation of human dignity in digital mental health (1). It is essential to recognize that while AI may supplement certain therapeutic functions, it cannot replace the embodied, relational, and intersubjective dimensions central to psychodynamic therapy.

By articulating what cannot be algorithmically replicated, we can better understand and preserve the essential core of psychodynamic practice while thoughtfully integrating technological advances where appropriate. This balanced approach will allow us to harness AI’s benefits without compromising the human connection at the heart of psychodynamic healing.

The future of psychodynamic work in an age of artificial intelligence requires not abandoning technology but rather clarifying with greater precision the uniquely human elements of therapeutic change. This clarity will guide us toward ethical and effective integration of AI in mental healthcare—one that enhances rather than replaces the profound human connections that make psychological healing possible.

## Data Availability

The original contributions presented in the study are included in the article/Supplementary Material. Further inquiries can be directed to the corresponding author.
